# Drug Repositioning to Accelerate Drug Development Using Social Media Data: Computational Study on Parkinson Disease

**DOI:** 10.2196/jmir.9646

**Published:** 2018-10-11

**Authors:** Mengnan Zhao, Christopher C Yang

**Affiliations:** 1 College of Computing and Informatics Drexel University Philadelphia, PA United States

**Keywords:** drug repositioning, Parkinson disease, heterogeneous network, social media

## Abstract

**Background:**

Due to the high cost and low success rate in new drug development, systematic drug repositioning methods are exploited to find new indications for existing drugs.

**Objective:**

We sought to propose a new computational drug repositioning method to identify repositioning drugs for Parkinson disease (PD).

**Methods:**

We developed a novel heterogeneous network mining repositioning method that constructed a 3-layer network of disease, drug, and adverse drug reaction and involved user-generated data from online health communities to identify potential candidate drugs for PD.

**Results:**

We identified 44 non-Parkinson drugs by using the proposed approach, with data collected from both pharmaceutical databases and online health communities. Based on the further literature analysis, we found literature evidence for 28 drugs.

**Conclusions:**

In summary, the proposed heterogeneous network mining repositioning approach is promising for identifying repositioning candidates for PD. It shows that adverse drug reactions are potential intermediaries to reveal relationships between disease and drug.

## Introduction

Parkinson disease (PD) is a chronic and progressive movement disorder with the main symptoms including tremor, slowed movement, rigidity, and postural instability. It is the second most common neurodegenerative disorder and affects more than 5 million people around the world. So far, the causes of PD remain unknown but are thought to be triggered by genetic or environmental factors that lead to the death of dopamine generating cells [1]. Current treatments and medications cannot reverse the effects of the disease but can manage the symptoms; however, they come with many serious adverse drug events. Therefore, it is important to develop new drugs for PD.

De novo drug development has been a prolonged process with very high development costs and a low success rate in the past decades. Meanwhile, drug repositioning, which identifies new indications for existing drugs, is suggested to be a more cost- and time-efficient strategy by the evidence. One main advantage is that since repositioning drugs have already been validated by pharmaceutical and toxicological tests, the time and cost for development and the risk of failure in early stages can be reduced markedly. Currently, systematic drug repositioning methods can be categorized as either disease-based, where discovery initiates from the clinical perspective of diseases, or drug-based, where discovery initiates from the chemical or pharmaceutical perspective of drugs [2]. Drug-based strategies are preferred when there is expertise or capability in using and understanding pharmacological properties of drugs or if rich pharmacological or chemical data for drugs are available. Disease-based strategies are preferred to overcome missing knowledge in the pharmacology of a drug or when repositioning efforts are to be focused on a specific disease or therapeutic category [3]. In this context, we proposed a disease-based approach to identify the repositioning drugs for diseases using social media data and pharmaceutical databases. Specifically, we connected drugs with their potential indications via adverse drug reactions (ADRs).

The phenome is emerging as a new source of information for drug repositioning because phenotypic information is shown to have great potential for detecting novel associations between drugs and diseases. Clinical side effects, which are capable of profiling drug-related phenotypic information, have been found to be helpful in discovering new therapeutic uses for drugs [4]. As a result, the ADR is becoming an important intermediary to connect drugs with diseases in drug repositioning because it reflects the physiological consequences and phenotypic expressions of the drugs. The rationale behind the ADR-based approaches of drug repositioning is based on the underlying mechanism of action linking ADRs with diseases when the ADR is shared by a number of drugs indicated for the disease [5].

Considering the huge potential of social media data for health care informatics, we collected user-generated content from an online health community as one data source. It has been proven that social media data provide a large volume of timely information contributed by health consumers, and the detection of adverse drug reactions using social media can achieve better results than detection using other traditional data sources such as spontaneous reporting systems or electronic health records [6-9]. As network-based approaches have been increasingly used to identify repositioning drug candidates in both drug-based and disease-based strategies [10-12] and heterogeneous networks can capture more essential and accurate features of the health care information compared with a homogeneous network, we exploited the heterogeneous networking mining methodology to reveal relationships between different medical entities.

## Methods

**Figure 1:**
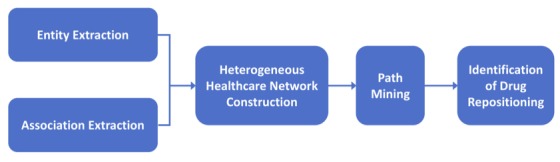
Workflow of drug repositioning for Parkinson disease based on adverse drug reactions.

Our method mainly comprises the following steps: (1) extracting ADR entities from user posts in online health communities; (2) computing association strength of ADR-drug, ADR-disease, and disease-drug pairs; (3) building heterogeneous networks based on association strength; and (4) identifying significant ADR-disease paths from the network and conducting drug repositioning. Figure 1 shows the workflow of our method.

### Adverse Drug Reaction Entity Extraction

A significant challenge existing in extracting ADR terms from social media data is the diverse expressions of adverse reactions. Health consumers describe their health issues using different vocabularies than medical professionals due to their lack of medical training. As a result, standard medical lexicons used by professionals like the US National Library of Medicine’s Unified Medical Language System are not applicable in analyzing health consumer–contributed content. To address this problem, we used the Consumer Health Vocabulary (CHV) Wiki [13] to build our ADR lexicon. The CHV Wiki links everyday health-related words to professional terms and jargon, and the goal is to bridge the communication gap between consumers and health care professionals [5]. It provides a list of preferred names of ADRs and the corresponding consumer-contributed expressions to each of them. For example, “anorexia” is a professional expression of an ADR, and the CHV Wiki extends it to “appetite lost,” “appetite loss,” “appetite lack,” “no appetite,” and several other common expressions. In our study, we used all of the expressions suggested by the CHV Wiki to extract ADR entities in user-generated information [14].

### Association Strength Calculation

After extracting ADR entities, we computed the association strength between ADR-disease, ADR-drug, and drug-disease pairs. In social media data, if 2 entities are mentioned together frequently, they are considered to be strongly associated [15]. We conducted co-occurrence analysis on those entity pairs to evaluate their association strength. In co-occurrence analysis, choosing proper analysis granularity is important. That means the co-occurrence analysis should be calculated within an appropriate scale. A post or comment might be set as an analysis unit, but they are usually very short and the users may jump into their problems without describing the context. Thus, a post or comment is too small an analysis unit to calculate the co-occurrence between entities. Instead, a thread, which contains a post and all the following comments, is more appropriate to be an analysis unit because it embodies all the discussions on a particular issue and contains all the keywords. Therefore, we calculated the co-occurrence frequency of entity pairs within each thread.

We applied association rule mining to determine the co-occurrence frequency between ADR-PD, ADR-drug, and drug-PD pairs. Mathematically, let *I*={*I*_1_, *I*_2_,..., *I*_*m*_} be a set of items and let *T*={*T*_1_, *T*_2_,…, *T*_n_} be a set of transactions, where each transaction is a subset of items such that *T*_*i*_⊆*I*. The association rule is an implication of the form *A* ⇒*B*, where *A* ⊂*I*, *B* ⊂*I*, and *A* ∩*B*=∅, which is referred to as an itemset. An itemset that contains *k* items is a *k*-itemset. Specifically, there are both 1-itemsets ({ADR}, {drug(R)}, {PD}) and 2-itemsets ({ADR, R}, {ADR, PD}, {R, PD}) in our experiments.

*Lift* is a commonly used measure based on probability theory in association rule mining. For instance, *lift* measures the strength of an association by considering not only the co-occurrence of *R* ∪*ADR* but also the correlation between itemsets {*R* } and {*ADR* }. To be specific, it computes the ratio of the proportion of threads containing both *R* and *ADR* above the expectation that *R* and *ADR* are independent of each other. The following shows the equations for computing *Lift* (*R* ⇒*ADR*):

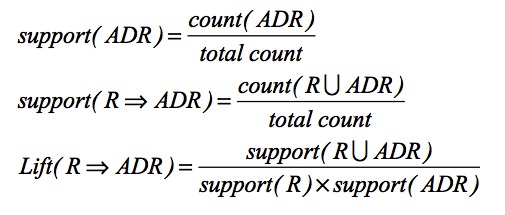



In which *count* (*ADR*) is the number of threads that contain target *ADR*, *count* (*R* ∪*ADR*) is the number of threads that contain both drug *R* and *ADR*, and *total count* is the total number of threads. Likewise, we can compute *Lift* (*D* ⇒*ADR*) and *Lift* (*D* ⇒*R*) with the same equation. The *lift* value is consistent with the strength of associations between 2 items.

### Heterogeneous Network Construction

A heterogeneous network is defined as a graph consisting of nodes connected by links, with at least 2 types of nodes and at least 2 types of links [16]. Let *N*={*n*_1_,*n*_2_,...,*n*_k_} be a set of nodes and *L*={*l*_1_,*l*_2_,...,*l*_*m*_} be a set of links, and *G*=(*N*, *L*) denotes the graph. In the graph *G*, each node *n*_*i*_∈*N* belongs to a particular type from *γ* and each link *l*_*i*_∈*L* belongs to a particular type from *τ*, where |*γ* |>1 or |*τ* |>1.

**Figure 2:**
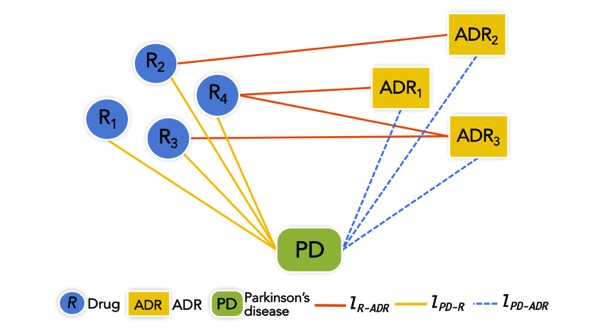
Heterogeneous health care network model.

In our heterogeneous health care network for PD, there are 3 types of nodes (drug, PD, and ADR) and 3 types of links (drug-ADR, PD-ADR, and drug-PD), that is, *γ*={*R*, *PD*, *ADR* } and *τ*={*L*_R-__ADR_, *L*_PD-__ADR_, *L*_PD-__R_}. Figure 2 presents a nondirectional graph model of the described heterogeneous health care network that is weighted, and the association of the 2 end nodes of a link determines the weight of the link. For instance, the weight of the link between a drug and an ADR is represented and computed by *Lift* (*R* ⇒*ADR*).

In addition, we predicted the strength of association between disease-ADR pairs through all possible paths suggested by the network in this work and proposed a path mining method: *Path* (*D-R-ADR*,*W*_D-__R_*W_R-__ADR_). ADR represents the harmful and unpleasant reactions of medicine use, and hence, the ADRs that are associated with a disease are highly influenced by the drugs that are used for the disease. We use drug as the bridge between disease and ADR. The weight of a path is computed by the products of *Lift* (*D* ⇒*R*) and *Lift* (*R* ⇒*ADR*).

The association between disease and ADR is then computed by the equation seen in below, in which *P* denotes all of the possible paths between *D* and *R*:

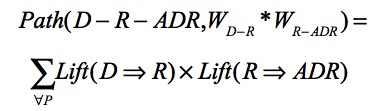



### Drug Repositioning Based on Disease–Adverse Drug Reaction Association

The basic hypothesis of drug repositioning is that if ADR *X* is significantly associated with disease *D*, then drugs that list *X* as a side effect should be evaluated as candidates for treating disease *D* [5]. For all the disease-ADR associations we detected, we first used a sample *t* test to determine whether they were significant and then applied drug repositioning to those significant associations.

The repositioning process is completed in 3 steps: (1) for each ADR contained in those significant ADR-disease associations, we consulted the Side Effect Resource (SIDER) database [17] to identify the drugs that list the identified ADR as a frequent side effect, (2) we directed those drugs to diseases via the drug-ADR-disease paths and obtained the final drug-disease associations, and (3) we removed the drug-disease pairs that have already been published in the Pharmacogenomics Knowledgebase (PharmGKB) [18]. Thus, the remaining drug-disease pairs could be considered as repositioning drug–disease associations and reported as the drug repositioning results.

## Results

### Data Collection

In our experiment, user-generated data were collected from MedHelp [19], one of the pioneers in online health communities. Since its introduction in 1994, MedHelp has been the exemplar in online health communities. Currently, it empowers over 12 million people each month to describe and discuss their health and medical problems and find answers. We collected data from MedHelp by implementing an automatic Web crawler that went through MedHelp webpages in HTML format page by page and retrieved information including user name, post, comment, and timestamp. The returned data were saved in .txt files and organized thread by thread, where each thread contained the original post and all the following comments. We collected 12,571 threads (containing 504,097 comments) by using Parkinson and the corresponding 55 drugs suggested by PharmGKB as query words on October 10, 2016. After extracting disease, drug, and ADR entities from the consumer-contributed content, we constructed a heterogeneous network containing 3 types of nodes and 3 types of links. In total, the network contained 255 nodes: 55 drugs, 199 ADRs, and 1 disease.

### Repositioning Drugs for Parkinson Disease

When the drugs treating a disease share a common ADR, the ADR and the disease are considered to be connected via an underlying mechanism of action. By applying the proposed heterogeneous network mining method, we detected the ADRs associated with PD and filtered the significant associations based on statistical analysis. As a result, we found 9 ADRs significantly associated with PD: muscle cramp, gastrointestinal disorder, nervous system disorder, angiopathy, somnolence, orthostatic hypotension, carpal tunnel syndrome, hallucination, and influenza.

We retrieved the drugs listing the ADRs but not indicated for PD from the SIDER database with several filtering conditions: the drugs indicate 1 of the 9 ADRs as “frequent,” “common,” or “postmarketing” or with an occurring percentage higher than 10. As a result, we identified 44 repositioning drugs for PD. Table 1 lists all the repositioning drugs, as well as whether these drugs are associated with PD based on literature analysis. By using drug names and Parkinson as queries in PubMed, we then investigated all the findings to understand the implications behind the associations of disease and repositioning drugs. We found evidence in PubMed literature to support a positive effect of some drugs on PD, evidence to support negative or adverse effects of some drugs on PD, evidence that some drugs exert both positive and negative effects on PD, and no explicit association between some drugs and PD.

Multimedia Appendix 1 presents the repositioning drugs that are supported by evidence from PubMed literature, and each row illustrates the drug and how it is labeled with the ADRs in the SIDER database. The second-to-last row total shows how many drugs were detected via the ADR, and the last row total shows how many drugs were only detected via the ADR.

Multimedia Appendix 1 indicates that nervous system disorder and gastrointestinal disorder are the 2 ADRs that contributed the most number of repositioning drugs, while the ADR carpal tunnel syndrome suggested no repositioning drug. Among the repositioning drugs that have evidence support, 54% (15/28) were detected via only 1 ADR and 46% (13/28) were detected via more than 1 ADR. Moreover, within the 13 drugs, 6 of them were detected via the ADR combination: nervous system disorder + gastrointestinal disorder, which suggests that nervous system disorder and gastrointestinal disorder might be a potential ADR combination for identifying repositioning drugs for PD. In addition, to investigate whether there is relationship between the number of ADRs and the number of PubMed sources, we did correlation analysis. The calculated correlation coefficient (Pearson *R*) approximately equals 0, indicating little correlation between those 2 factors.

### Analysis of the Repositioning Drugs Based on Literature

Table 2 summarizes the drugs analyzed in this section and their original labeled indications.

**Table 1:** Repositioning drugs and literature evidence.

Drug	Positive evidence	Negative evidence	No evidence
amphotericin B	✓	✓	
bupropion	✓		
carbamazepine	✓		
citalopram	✓	✓	
clomipramine	✓		
diltiazem		✓	
donepezil	✓		
fluoxetine	✓		
fluvoxamine	✓		
gabapentin	✓	✓	
glatiramer acetate	✓		
lamotrigine	✓	✓	
levetiracetam	✓		
methylphenidate	✓		
mirtazapine	✓		
modafinil	✓		
nefazodone	✓		
oxcarbazepine	✓		
paroxetine	✓		
phenytoin	✓		
rivastigmine	✓		
salbutamol	✓		
sertraline	✓		
thalidomide	✓		
topiramate	✓		
tramadol		✓	
valproic acid	✓		
ziprasidone	✓	✓	
alprazolam			✓
budesonide			✓
clarithromycin			✓
granisetron			✓
lansoprazole			✓
levonorgestrel			✓
mefloquine			✓
mycophenolic acid			✓
omeprazole			✓
tacrolimus			✓
tiagabine			✓
tobramycin			✓
topotecan			✓
valganciclovir			✓
venlafaxine			✓
vigabatrin			✓

**Table 2:** Repositioning drugs and their labeled indications.

Labeled indication	Drug name
Immunomodulatory	Thalidomide
Multiple sclerosis	Glatiramer acetate
Asthma	Salbutamol
Depression	Bupropion, citalopram, clomipramine, paroxetine, sertraline, fluoxetine, fluvoxamine, mirtazapine, nefazodone
Alzheimer disease	Donepezil, rivastigmine
Epilepsy	Carbamazepine, gabapentin, lamotrigine, levetiracetam, topiramate, valproic acid, oxcarbazepine, phenytoin
Fatigue	Methylphenidate, modafinil

#### Thalidomide

Thalidomide is an immunomodulatory drug used mainly for certain cancers. The studies using mice showed that thalidomide improved the neurotoxicity induced by 1-methyl-4-phenyl- 1,2,3,6-tetrahydropyridine as seen by a significant increase of dopamine, suggesting that thalidomide can improve the functional damage of the nigrostriatal cell substratum by the production of dopamine [20]. The neuroprotective effect makes thalidomide a potential adjuvant medication for PD. In addition, lenalidomide, a thalidomide derivative, was found to reduce motor behavioral deficits and improve dopaminergic fiber loss in the striatum by reducing microgliosis in both the striatum and hippocampus. This finding supports the potential of lenalidomide to address maladaptive neuroinflammation in PD [21].

#### Glatiramer Acetate

Conventional treatments for PD mainly address the dopamine deficiency, but some postmortem studies have showed that brain-derived neurotrophic factor (BDNF) deficiency may also play a role in PD pathogenesis, and this is supported by the finding that BDNF therapy is effective in animal models of PD [22]. Glatiramer acetate is an immunotherapy drug approved for treating multiple sclerosis. As glatiramer acetate can enhance central BDNF activity and augment neurogenesis, it may be useful to address the BDNF deficiency in PD; by exerting an anti-inflammatory effect, it can address the inflammatory process in the brain associated with PD [23]. According to this evidence, glatiramer acetate can be a potential medication for PD.

#### Salbutamol

Salbutamol is mainly used for asthma, bronchitis, emphysema, or other bronchospasms in lung diseases. There are preliminary studies showing that PD patients who were given salbutamol as adjunctive therapy improved in response to levodopa, as salbutamol enhances transport of levodopa across the blood-brain barrier [24]. An open-label pilot study also suggested that muscle mass and therapeutic response to levodopa in PD patients with fluctuation were improved by salbutamol [25]. To confirm its effects on PD, further studies such as double-blind and placebo-controlled studies are needed.

#### Antidepressants

Beside the motor dysfunctions in PD, the occurrence of nonmotor symptoms, such as psychological disorders, is very high. Specifically, depression occurs in 20% to 50% of PD patients, associated with increasing disability. Some antidepressants have been used in PD, but few studies have been conducted to support their efficacy and investigate adverse effects for PD patients. Therefore, an adequate therapeutic answer for treating depression together with PD is needed. Among the identified repositioning drugs, several are antidepressants.

##### Bupropion

Dopamine agonists have been considered as antidepressants in some studies, but they may cause side effects such as confusion, somnolence, and dizziness, so the role of the dopamine agonist in depressive PD still needs to be explored [26]. Bupropion, as a dopaminergic and noradrenergic antidepressant, could be a possible treatment for depressive symptoms associated with PD without serotonin-related side effects. Moreover, depression was improved in 5 clinical reports when using bupropion on PD patients while treatments with several other antidepressants were unsuccessful [27]. For instance, panic disorder was improved markedly on a 57-year-old female PD patient after introducing bupropion [28].

##### Citalopram

Citalopram is an antidepressant belonging to a group of drugs called selective serotonin reuptake inhibitors. As recent research indicates that selective noradrenergic (atomoxetine) and serotonergic (citalopram) reuptake inhibitors show potential to improve response inhibition on some PD patients, a double-blind experiment was conducted to investigate the behavioral efficacy of citalopram and atomoxetine [29]. Results supported the hypothesis that they are effective in inhibitory control. However, a retrospective survey showed that citalopram may trigger acute dystonia and exacerbate the abnormal movements of PD [30]. Therefore, the efficacy or adverse effect of citalopram for PD needs to be explored further.

##### Clomipramine

Clomipramine is a tricyclic antidepressant used to treat obsessive-compulsive disorder by increasing the activities of certain chemicals in brain. It was reported that delusions and hallucination conditions were improved by using clomipramine on a PD patient with depression [31].

##### Paroxetine

Through a 12-week placebo-controlled clinical experiment, paroxetine was found to improve the affective symptoms, somatic symptoms, and cognitive symptoms in depressed PD patients [32].

We did a literature analysis on the other 5 antidepressants (sertraline, fluoxetine, fluvoxamine, mirtazapine, and nefazodone) as well and found that those drugs are effective in dealing with the depression of PD patients. As the studies mostly discuss the effectiveness of the antidepressants on depression of PD patients rather than on PD itself only, we did not present all of the details here.

#### Medications for Alzheimer Disease

##### Donepezil

Donepezil, a cholinesterase inhibitor that works by increasing the amount of acetylcholine in the brain to reduce dementia symptoms, is indicated for Alzheimer disease. An exploratory study of 9 patients was conducted to study the effects of donepezil in PD patients with dementia, especially the effects of dose escalation [33], and the results showed that certain dose escalations of donepezil are useful for patients in the long term. In addition, the results of a randomized double-blind study showed that the combined use of donepezil and Di-Huang-Yi-Zhi is very effective in treating PD dementia, with a possible underlying reason that problems with the cholinergic system were ameliorated by such a combination therapy [34].

##### Rivastigmine

Rivastigmine is a drug used to treat Alzheimer disease and proven to be effective for mild cognitive impairment in PD. A double-blind placebo-controlled clinical study showed that a global rating of cognition, health status, and anxiety severity as well as the cognitive abilities according to a performance-based measure were improved by using rivastigmine [35].

#### Antiepilepsy Medications

##### Gabapentin

Gabapentin is an antiepileptic drug that affects the chemicals and nerves that cause seizures and pain to treat epilepsy and some types of nerve pain. Common medications for treating visual hallucination and the decrease of dopamine agonists often cause an exacerbation of motor symptoms, while gabapentin can directly affect the glutamic acid neuron system and the γ-amino butyric acid neuron system as an antiepilepsy drug. In a case report, both visual hallucination and pain of a PD patient were alleviated by using gabapentin, without any adverse effects [36]. However, in several cases, dyskinesia and bilateral ballism were induced when using gabapentin in PD diseases. Further studies are needed to prove the efficacy of this drug.

##### Lamotrigine

A group of researchers did both in vivo and in vitro experiments to examine whether safinamide (monoamine oxidase B and sodium channel blocker) has effects on microglial activation and the degeneration of dopaminergic neurons, which are closely associated with PD [37]. Results showed that safinamide has positive effects on microglial activation and protects dopaminergic neurons from degeneration in the 6-hydroxydopamine model of PD. In the experiments, rasagiline, a monoamine oxidase B inhibitor, and lamotrigine, a sodium channel–blocking drug, both exerted the protection of dopaminergic neurons, suggesting that safinamide can function in either or both mechanisms. In addition, the usefulness of lamotrigine in PD has been proven in several other studies, including a double-blind study on 20 patients [38] and an in vivo experiment on mice [39].

##### Levetiracetam

Levetiracetam was proved to be useful for the levodopa-induced dyskinesias in PD in a double-blind, placebo-controlled crossover trial [40].

##### Topiramate

Topiramate is mainly used to treat seizures in certain patients and sometimes used to treat migraine headaches. As it reduced levodopa-induced dyskinesia in animal models without affecting PD symptoms, a randomized, double-blind, placebo-controlled trial was then conducted on 13 patients. However, the results showed that topiramate worsened dyskinesia in PD patients [41].

##### Valproic Acid

Valproic acid is used for treating epilepsy by affecting chemicals that cause seizures [42]. An in vitro model was applied to investigate the treatment of valproic acid on synaptic loss and the underlying molecular mechanism. The experiment showed that the synaptic damage induced by amyloid-β, which is associated with Alzheimer disease, and by another neurodegenerative-associated protein, α-synuclein, which is associated with PD, could be reduced and protected by valproic acid [43], as valproic acid can inhibit the aberrant activation of amyloid-β–dependent cytoplasmic phospholipase A2. Therefore, valproic acid may be a potential therapy for Alzheimer disease and PD [44].

The other 2 antiepilepsy drugs that were found to be useful for PD are oxcarbazepine and phenytoin. Studies were conducted to evaluate the effectiveness of using antiepileptic drugs (eg, oxcarbazepine) together with some 3-hydroxy-3-methylglutaryl– coenzyme A reductase inhibitors for prevention of PD and other neurological diseases, and the experiments showed that the dopaminergic effect of oxcarbazepine is useful in the treatment of PD [45]. Another study, however, suggests that oxcarbazepine should be used with care because of its possible psychiatric side effects [46]. Epilepsy and PD often co-occur in the elderly, and phenytoin is used to control certain types of epilepsy, so many PD patients take phenytoin. Studies have been conducted to observe PD patients taking phenytoin, and a relationship is suspected between the function of phenytoin and the pathogenesis of PD [47].

#### Drugs Originally Used for Fatigue

##### Methylphenidate

Methylphenidate is a central nervous system stimulant used for hyperactivity disorder, found to be useful to address the maladaptive behavioral or cognitive aspects of fatigue in Parkinson patients [48].

##### Modafinil

Modafinil is a wakefulness-promoting agent used for sleep disorder and an effective treatment for fatigue in PD [49].

#### Other Drugs Known to Cause Adverse Effects in Parkinson Patients

##### Amphotericin B

Amphotericin B is used for treating progressive and potentially life-threatening fungal infections, but it was found to have interactions with PD in some clinical reports. There are several reports of neurotoxicity being caused by the increasing use of intrathecal amphotericin B—for instance, transient signs of parkinsonism occurred in 1 patient receiving the drug for cryptococcal meningitis [50] and in 3 children receiving it for pulmonary aspergillosis or sinus aspergillosis [51]. Although the indications of amphotericin B do not involve the nervous system, it was thought to have a direct toxic effect on the nervous tissue.

##### Diltiazem

Diltiazem is a calcium channel blocker that can relax the muscles of heart and blood vessels and is mainly used for hypertension and angina. However, it was found to induce Parkinson syndrome in some patients [52-53].

##### Tramadol

Tramadol is an analgesic widely prescribed because of its low abuse potential, but researchers found serotonin syndrome, a life-threatening adverse reaction, in a PD patient who used tramadol and ziprasidone [54].

##### Ziprasidone

Ziprasidone belongs to the class of atypical antipsychotics and is used for central or mood disorders. A clinical trial that conducted parallel comparison experiments on 14 patients demonstrated that ziprasidone is effective in ameliorating psychotic symptoms in PD patients [55]. However, serotonin syndrome occurred in a PD patient using ziprasidone for bipolar disorder [54]. Therefore, further studies are needed to evaluate the efficacy of ziprasidone for PD.

## Discussion

### Principal Findings

In drug repositioning research, results are only suggestions or predictions rather than confirmed truth until clinical trials are conducted. As a result, the evaluation of method performance becomes a difficult task, because there is no gold standard. In previous studies, there have been two common ways of evaluation: computational assessment, based on the co-occurrence of drug-disease terms in biomedical literature, and experimental assessment, based on in silico or in vitro experiments. Here we have adopted the computational assessment but beyond counting the co-occurrence frequency, we conducted a literature review to investigate whether the scientific studies in the published articles supported the repositioning of the drug to PD. In the future, experimental assessment could be exploited to reinforce the evaluation.

Among the discovered repositioning drugs, some have the potential to treat PD via their neuroprotection or neurotoxicity prevention function, while several others show the effects in clinical practice without the underlying mechanism being known; therefore, exhaustive analysis is needed of the drugs. In addition, we identified several psychological drugs that are proven to be effective without inducing adverse effects when used to treat PD. As psychological symptoms occur in a high ratio of PD patients, these findings are quite useful. Based on the results, the repositioning drugs that have positive functions on the neural system are the most promising findings and are the primary candidates for further experimental assessment. Meanwhile, the repositioning findings that are commonly used for other complicating diseases on PD patients should be tested more carefully for efficacy and risk evaluation.

In this work, we relied on the rational association between disease and ADR to find potential repositioning drugs and achieved an appreciable result. To investigate whether there is any relationship between the number of intermediary ADRs and the number of supporting literature sources, we did a correlation analysis, but there is little correlation between the 2 factors according to the calculated Pearson *R* coefficient (≈0). A possible reason could be the size of our dataset (eg, ADR and drug). In a future study, we can expand the dataset size to include more ADRs and involve more biomedical characteristics, such as chemical or biological characteristics or a combination of the two, hoping for better performance.

### Conclusions

In this study, we proposed a computational drug repositioning method based on a heterogeneous network and used the rational association between disease and ADR to reveal repositioning drugs. Experiment results demonstrated that our approach is able to identify FDA-approved novel drugs for PD, and many of the predictions are supported by existing studies according to the literature-based investigation. Although further studies are needed to confirm the pharmaceutical effects of these repositioning drugs for PD, this study suggests the possibility and effectiveness of applying systematic drug repositioning methods to drug development.

## Appendix

Multimedia Appendix 1 Repositioning drugs detected via each adverse drug reaction.

## Abbreviations

ADR: adverse drug reaction
BDNF: brain-derived neurotrophic factor
CHV: Consumer Health Vocabulary
PD: Parkinson disease
PharmGKB: Pharmacogenomics Knowledgebase
SIDER: Side Effect Resource database
