# Supplementary material for: Drug Repositioning to Accelerate Drug Development Using Social Media Data: Computational Study on Parkinson Disease
Source: J Med Internet Res. 2018 Oct 11;20(10):e271. doi: 10.2196/jmir.9646 (PMC6231748; doi:10.2196/jmir.9646)
Supplement: Multimedia Appendix 1 [file jmir_v20i10e271_app1.pdf]

Multimedia Appendix 1. Repositioning drugs detected via each ADR.

		Cramp muscle	Gastrointestinal disorder	Nervous system disorder	Angiopathy	Somnolence	Orthostatic hypotension	Carpal tunnel syndrome	Hallucination	Influenza	Num-ADR	Num – PubMed Article
1	**amphotericin b**				P						1	5
2	**bupropion**			10							1	4
3	**carbamazepine**			C							1	7
4	**citalopram**	F		P							2	9
5	**clomipramine**								C		1	3
6	**diltiazem**						P				1	15
7	**donepezil**									F	1	27
8	**fluoxetine**		C								1	13
9	**fluvoxamine**					C					1	4
10	**gabapentin**			P							1	11
11	**glatiramer acetate**			P							1	7
12	**lamotrigine**					10					1	22
13	**levetiracetam**		10	P							2	21
14	**methylphenidate**		10	10							2	20
15	**mirtazapine**		P	P							2	5
16	**modafinil**		P	P							2	10
17	**nefazodone**				P						1	3
18	**oxcarbazepine**					C					1	1
19	**paroxetine**		C	P							2	9
20	**phenytoin**					10					1	3
21	**rivastigmine**						F			F	2	32
22	**salbutamol**	P									1	1
23	**sertraline**		P	P							2	5
24	**thalidomide**		10	C		10					3	10
25	**topiramate**		P	P		10					3	2
26	**tramadol**			P	P		P		P		4	6
27	**valproic acid**					10				10	2	17
28	**ziprasidone**		P	P	P	10	P			F	6	8
	**Total**	2	10	15	4	8	4	0	2	4		
	**Total-only**	1	1	4	2	4	1	0	1	1		

a. P: postmarketing, C: common, F: frequent, 10: frequency >= 10%

b. Number in column “Num-ADR” denotes the number of ADRs that suggest the drug

c. Number in row Total denotes the number of drugs detected by the heading ADR in total

d. Number in row Total-only denotes the number of drugs detected only by the heading ADR in total
